# Fermented Citrus Lemon Reduces Liver Injury Induced by Carbon Tetrachloride in Rats

**DOI:** 10.1155/2018/6546808

**Published:** 2018-05-20

**Authors:** Yi Jinn Lillian Chen, Pei-Chi Chou, Chang Lu Hsu, Jeng-Fung Hung, Yang-Chang Wu, Jaung-Geng Lin

**Affiliations:** ^1^Graduate Institute of Chinese Medicine, China Medical University, Taichung 40402, Taiwan; ^2^Graduate Institute of Science Education & Environmental Education, National Kaohsiung Normal University, Kaohsiung 80201, Taiwan; ^3^Research Center for Natural Products & Drug Development, Kaohsiung Medical University, Kaohsiung 80708, Taiwan

## Abstract

Fermented lemon juice displays a variety of important biological activities, including anti-inflammatory and antioxidant capabilities. The aim of the present study is to investigate hepatic-protective effects of no-sugar-added fermented lemon juice (FLJ) for liver inflammation caused by carbon tetrachloride (CCl_4_) in rats. Rats are divided into six groups: H_2_O, CCl_4_ + H_2_O, CCl_4_ + silymarin, and CCl_4_ plus three different FLJ doses by oral administration, respectively. The results show that the contents of plasma ALT and AST, hepatic lipid peroxidation, splenomegaly, and liver water are reduced significantly in rats under FLJ treatment, and pathological examination of liver fibrosis is improved. The reduced hepatic injury by increasing liver soluble protein and glutathione and albumin is observed in FLJ treated groups, and FLJ has comparable efficacies to medicine silymarin in liver therapies. The no-sugar-added FLJ differs from traditional fermentation by adding lots of sugar and prevents any hidden sugar intake while taking it as a complimentary treatment for liver inflammation. The green color and the taste of sourness are both associated with treating and healing the liver based on the five-element theory in traditional Chinese medicine, and the green and sour FLJ may be applied to the ancient theory in preventing hepatic injury accordingly.

## 1. Introduction

Liver cancer is one of the most extensive cancers worldwide [1]; the liver is a silent organ, where preventive approaches are necessary if prevention can be achieved in our daily diets. Hesperidin and hesperetin, its aglycone, are both flavonoids (phytochemicals), which are plentiful in citrus fruits, such as lemons, oranges, and grapefruits [2]. Citrus flavonoids are also used effectively in complementary therapies, as it possesses antioxidant, anti-inflammatory, anticancer, and pharmacological properties [3, 4]. Some investigations indicate that lemon ethanol extract is hepatoprotective both in vivo and in vitro for liver injury induced by carbon tetrachloride (CCl_4_) [5]. Hesperidin, a pure compound from citrus species, has protective abilities against liver injury, and the results are positive [6]. Citrus lemon fruits, commonly known as lemon, are one of the most important fruits cultivated in the southern part of Taiwan, and due to the warm weather, it is always consumed in a green color. In traditional medicine, the sour taste and the green color are related to the liver. Lemon, the green and sour fruit, is selected to evaluate its anti-inflammatory efficacies in hepatic injury in this study.

The Dietary Guidelines for Americans 2010 recommend increasing vegetable and fruit consumption but reducing sugar intake and also eating different colors, such like green, red, and orange [7]. Vegetables, fruits, and beans are preserved with extra added sugars as so-called healthy foods. In order to follow the dietary guidelines, preparing food with no sugar added as nutrient-dense foods [8] is an objective in this study.

Lemon is known as a healthy fruit, but it is usually drunk with lots of sugar or preserved by adding extra sugar, which causes a sugar consumption issue. A new fermentation method of lemon fruits by adding no sugar but only yeasts is designed to meet the challenge of requisitions of the dietary guidelines to avoid people having a healthy fermented diet but drinking too much sugary beverages unintentionally. Some studies indicate that drinking sugar-sweetened beverages may cause poor health [9].

Hepatotoxicants, such as CCl_4_, are used mostly to express oxidative stress, free radicals, and histological damage in the liver [10]. In this study, no-sugar-added fermented lemon juice (FLJ) is taken to evaluate its anti-inflammation efficacies of CCl_4_-induced liver damage, due to the fact that its figures of ORAC (oxygen radical absorption capacity) and total phenol are much higher than lemon juice after fermentation. We examine the hepatoprotective activities and pharmacological effects of FLJ on CCl_4_-induced toxicity of liver in rats. The FLJ with 3 doses (5 mL/kg, 10 mL/kg, and 26 mL/kg) is designed to compare with 3 other groups: the control (without CCl_4_) group, CCl_4_-induced + H_2_O, and CCl_4_-induced + silymarin treatment groups. Silymarin is the drug for the treatment of toxic liver disease which has been used for over 20 years in clinical practice [11].

## 2. Materials and Methods

### 2.1. Preparation of Lemon Juice and Fermented Lemon Juice

Organic green lemons from the southern part of Taiwan were supplied by contracted farmers; after washing, lemon juice is extracted by squeezing lemons with the peel and seed. Preparing FLJ: the extracted lemon juice is implanted with cultivated yeasts (organic lemon and yeasts DMS32004 and DMS32005 used are supplied by Jian Mao Biotechnology Co. Ltd., Taiwan, in fermenting lemon as remedies in this study). The yeast concentration is 5 × 10^6^ FU/mL~10^7^ CFU/mL, and fermentation is conducted at 28°C at pH 2.3. After 21 days' fermentation process, the fermented lemon juice is sterilized at 90°C for 15 minutes; then, the final fermented lemon juice (FLJ) is sealed and stored at room temperature for further usage.

Carbon tetrachloride (CCl_4_) is an effective hepatotoxic chemical that produces free radicals and is widely used to induce acute hepatic injury in experimental animal models [12]. In this study, the fermented lemon juice is prepared as remedies to feed to the hepatic injured Wistar rat induced by CCl_4_ effectives of hepatoprotective experiment.

### 2.2. Remedies Design

For this study, the FLJ is given orally in different doses. Normally, for an adult, it is suggested to take 50 mL of FLJ per day. According to the calculation of metabolic rate from rats to humans, which is about 6.2 times, the dose for the rat is calculated as 50 mL/60 kg (human weight) 6.2 = 5.2 mL/kg. The doses are designed to be given orally at 3 different dosages: 5 mL/kg, 10 mL/kg (2 times the human dose), and 26 mL/k (5 times the human dose) per day. The control group is given only purified water by the same designed doses. In rats weighing 100 g, the dose of 0.5 mL (low dose group) or 1.0 mL (middle dose group) is given. The vacuum-concentrated FLJ is given to the highest dose group; the dose is 1.0 mL/100 g. The concentration of FLJ of 2.6 times is prepared for the high dosage group, which means 26 mL is concentrated into 10 mL.

### 2.3. Animal Care

A total of 72 male laboratory Wistar rats were purchased from BioLASCO Co. Ltd. (Taipei, Taiwan). Animals were housed in China Medical University Animal Room at rat cages, under a 12 h light/12 h dark cycle (at 8 a.m. light on and at 8 p.m. light off), in an air-conditioned room at a temperature of 22 ± 2°C. After one week, healthy rats were selected for the experiments. Food (Prolab RMH 2500) and purified water were provided ad libitum. Rats are equally divided randomly into 6 groups: control group (10 rats) and other groups that are all with 12 rats; the smallest 2 rats were excluded. All animals received good care and the study complied with the guidelines of China Medical University for the practices on laboratory animals.

### 2.4. CCl_4_-Induced Liver Injury

Liver injury is induced in 5 groups of 12 rats by oral administration of CCl_4_ in the morning at 8:00 a.m. to 8:40 a.m. twice a week for 8 weeks. The CCl_4_ is diluted 1 : 4 in olive oil as 20% diluted oral remedies and the dosage is 0.2 mL/100 g rat each time. The rats received no CCl_4_, CCl_4_ + H_2_O, or CCl_4_ + FLJ and CCl_4_ + silymarin.

After the rats received CCl_4_ for one week and then at three weeks and six weeks, the blood was withdrawn from their tails under isoflurane inhalation anesthesia. In 8 weeks' time, after blood was drawn from the rats, the rats were sacrificed at the same time and the liver and spleen were quickly removed. The organs were weighed after they were washed clear with cold normal saline and sucked up dry. The largest lobe of the liver is divided into two parts. One part is soaked with 10% neutral formalin for preparation of the pathological slices. After weighing, a second part is completely dried at 100°C for examination of collagen contents [13]. The remaining parts are stored in 4 packs at −80°C for further use.

### 2.5. Assay of Blood Plasma ATL and AST

Blood is centrifuged at 4,700 rpm for 15 min to separate plasma. In week 1, week 3, week 6, and week 8, the plasma alanine aminotransferase (ALT), asparate aminotransferase (AST), and albumin (week 8 only) are assayed using clinical test kits (Roche Diagnostics, Rotkreuz, Switzerland) on a spectrophotometric analyzer (Cobas Mira Plus, Roche, Rotkreuz, Switzerland) [13].

### 2.6. Assay of Glutathione

GSH in liver homogenates is assayed by measuring the fluorescence produced by* O*-phthalaldehyde at 420 nm, by the method of Hissin and Hilf [14]. The absorbance of the fluorescence of emitted light wavelengths is set to 350 and 420 nm, respectively [15]. It is expressed as *μ*mol/g tissue.

### 2.7. Assay of Hepatic Lipid Peroxidation

The homogenate is used for the determination of lipid peroxidation levels according to the method of Ohkawa et al. [16], using 1.15% KCl, in 10% liver tissues, and is centrifuged at 4,000 rpm for 5 min, followed by using 2-thiobarbituric acid for evaluation. Lipid peroxidation is expressed as the amount of malondialdehyde (MDA) mg/g protein.

### 2.8. Assay of Liver Protein

Liver protein is estimated using liver tissues visualized by Coomassie Blue (Kenlor Industries, Inc., USA), and the absorbance is measured at 540 nm. The amount of protein is expressed in* mg/g*.

### 2.9. Assay of Liver Hydroxyproline and Liver Water

Hydroxyproline is assayed according to the method of Neuman and Logan [17]. Dried liver tissue after hydrolysis is oxidized by H_2_O_2_ and* p*-dimethylaminobenzaldehyde and its absorbance is measured at 540 nm. The amount of hydroxyproline is expressed in* μg/g tissue*. Liver water is weighed by the weight of the liver tissue of hydroxyproline minus dried-out liver tissue, and the differences in the weight of the liver water are expressed in %.

### 2.10. Assay of Activities of SOD, Catalase, and Glutathione Peroxidase (GSH-Px)

SOD activity is assessed according to the method of Xia et al. [18], using a Ransod kit (Randox Lab., UK). This method is based on the formation of red formazan by the reaction of 2-(4-iodophenyl)-3-(4-nitrophenyl)-3-(4-nitrophenol)-5-phenyltetrazolium chloride and the superoxide radical. One unit (U) of SOD is evaluated as the amount of enzyme to inhibit the reduction of formazan by 50%, and the results are expressed in U/mg protein.

Catalase activity is measured according to the method of Aebi [19]. The rate of reduction is the measurement of catalase activity, and one unit of enzyme activity is defined as the amount of enzyme shown as *K* which is expressed in U/mg protein.

Glutathione peroxidase (GSH-Px) activity is assessed using a Ransel kit (Randox Lab., UK) according to the method of Xia et al. [18]. The GSH-Px activity is defined as every 1 min the enzyme required to process 1 *μ*mol of NADPH to NADP as 1 U, and the results are expressed in mU/mg protein.

### 2.11. Pathological Examination

After fixation of the liver tissue with formalin, tissue samples were wrapped and sliced following standard procedures and stained with H&E stain (hematoxylin-eosin stain) and Sirius Red stain. Liver injury is scored in gradings (from 0 to 4 grades) [20].

### 2.12. Statistics Analysis

The results are expressed as mean ± SD. All experimental data are analyzed using one-way analysis of variance, and also liver histopathological examination data are analyzed by Duncan's multiple range test; *P* value < 0.05 is considered statistically significant.

## 3. Results

### 3.1. Weight Changes

After inducing CCl_4_ (0.2 mL/100 g body weight; diluted 1 : 4 in olive oil) in 5 groups of 12 rats in each group by oral administration twice a week for 8 weeks' time, the weights of the rats from the control group (without CCl_4_) and the rats with CCl_4_ are compared. As shown in Table 1, the rats without CCl_4_ (control group) are heavier than the rats that were induced by CCl_4_. Among the CCl_4_ groups, CCl_4_ with FLJ (5 mL, 10 mL, and 26 mL doses), CCl_4_ with silymarin, and CCl_4_+ H_2_O, the weights are similar.

### 3.2. Effects of Plasma ALT, AST, and Albumin

As shown in Table 2, for rats with CCl_4_ treatments (at weeks 1, 3, 6, and 8), the results in plasma AST and ALT activities are obviously higher than in the control group. In week 1, in oral treatments of FLJ (in 5 mL and 10 mL dose groups) and silymarin, the plasma AST and ALT activities are lower than in the CCl_4_ + H_2_O group. However, the activities of plasma AST and ALT from CCl_4_ + FLJ (26 mL dose group) and CCl_4_ + H_2_O are not different. In week 3, the activities of plasma AST indicate that FLJ (5 mL, 10 mL, and 26 mL doses) and silymarin treatment groups are lower than CCl_4_ + H_2_O groups meaningfully. The plasma ALT activities in FLJ low dose and silymarin groups are lower than in CCl_4_ + H_2_O obviously, and in FLJ (10 mL and 26 mL doses group) and CCl_4_ + H_2_O group, there are no significant differences. In weeks 6 and 8, in the CCl_4_ induced with treatments of FLJ (5 mL, 10 mL, and 26 mL doses) and silymarin groups, the plasma AST and ALT are lower than in the CCl_4_ + H_2_O obviously. In week 8, the data of albumin levels in the CCl_4_ + H_2_O group are lower than the control group.

### 3.3. Effects of Liver and Spleen Weights

After CCl_4_ treatments induced liver injury, hepatic weights (weight and relative weights) among the CCl_4_ treated groups were heavier than the control group (Table 3). The liver weights from FLJ (10 mL and 26 mL doses) groups and silymarin group are lower than of CCl_4_ + H_2_O, and between the FLJ (5 mL dose) group and the CCl_4_ + H_2_O group, there are no differences.

In CCl_4_-induced rats, their percentages of liver water are higher than the control group; and after treating with FLJ (5 mL, 10 mL, and 26 mL doses), their percentages of liver water were lower than in the CCl_4_ + H_2_O group. The silymarin group has no big differences after comparing with CCl_4_ + H_2_O group.

The weights (weight and relative weights) of spleen in the CCl_4_ treated groups are much heavier than in the control group (Table 4). The increase in spleen weights caused by CCl_4_ is significantly reduced by FLJ (5 mL, 10 mL, and 26 mL doses) and silymarin treatments.

### 3.4. Effects of Glutathione, Malondialdehyde, Protein, and Hydroxyproline

In CCl_4_-induced liver injury in rats, the glutathione contents in the liver decrease. After treating with FLJ (5 mL, 10 mL, and 26 mL doses) or silymarin, the contents of glutathione were higher than in the CCl_4_ + H_2_O group. In rats with liver injury caused by CCl_4_, the malondialdehyde contents in the liver increased and the lipid peroxidation also increased apparently. In the FLJ (5 mL, 10 mL, and 26 mL doses) and silymarin treated groups, the contents of malondialdehyde became lower than in the CCl_4_ + H_2_O group (Table 5).

In rats with CCl_4_-induced liver injury, the soluble protein contents in the liver decreased. In the FLJ (26 mL dose) treated group, the contents of soluble protein turned out to be higher than the CCl_4_ + H_2_O group. In the FLJ (5 mL and 10 mL doses) and silymarin treated groups, there were no obvious differences in the liver protein contents from the CCl_4_ + H_2_O group. In rats with CCl_4_-induced liver injury, the hydroxyproline contents in the liver increased apparently. In FLJ (5 mL, 10 mL, and 26 mL doses) and silymarin treated groups, the hydroxyproline contents were lower than in the CCl_4_ + H_2_O group (Table 6).

### 3.5. Effects of Enzyme Activities of SOD, Catalase, and GSH-Px Antioxidant

In this study, as shown in Table 7, in rats with CCl_4_-induced liver injury, the activities of 3 antioxidant enzymes—SOD, catalase, and GSH-Px—were significant lower than in the control group. In FLJ (5 mL, 10 mL, and 26 mL doses) and also silymarin treated groups, the activities of enzymes SOD and catalase are not different from the CCl_4_ + H_2_O group. In the FLJ (5 mL, 10 mL, and 26 mL doses) treated groups, the GSH-Px activities are similar to the CCl_4_ + H_2_O group. In the silymarin treated group, the activities of GSH-Px are higher than in the CCl_4_ + H_2_O group.

### 3.6. Effects of Pathological Changes

**Figure 1:**
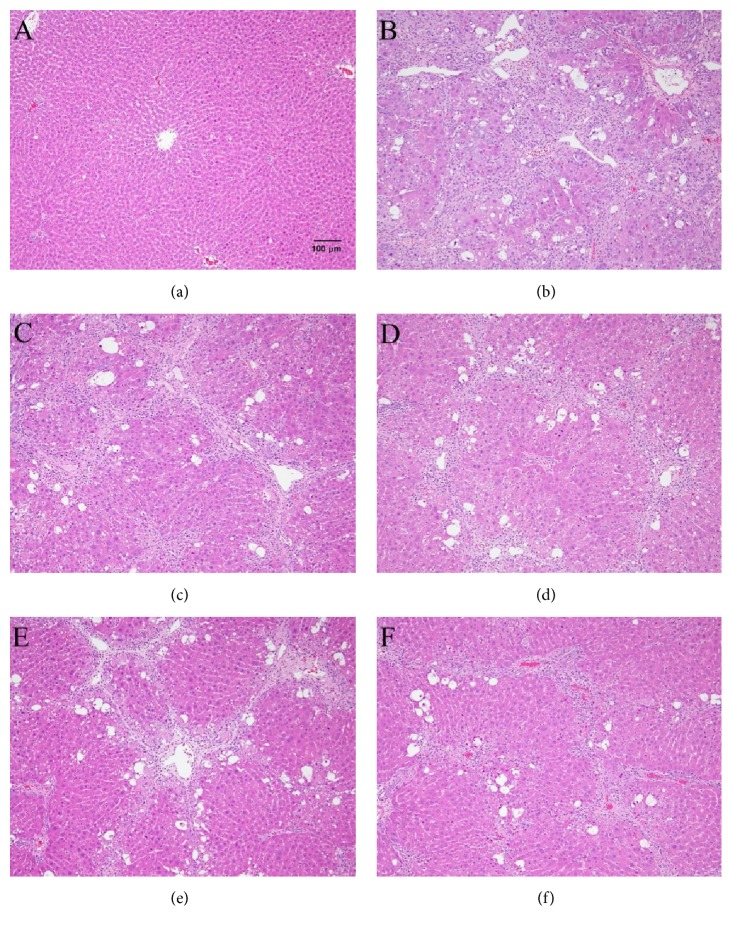
Treatment with FLJ improved the histology of CCl_4_ treated rat liver (H&E stain). H&E staining: (a) control group and (b) the group that received CCl_4_. Note that fatty changes and necrosis are observed; (c) CCl_4_ + FLJ 5 mL/kg group; (d) CCl_4_ + FLJ 10 mL/kg; (e) CCl_4_ + FLJ 26 mL/kg; (f) CCl_4_ + silymarin 200 mg/kg.

As Tables 8 and 9 and Figure 1 show, in the livers of CCl_4_-induced rats, the vacuolization and necrosis of the liver are obviously stained by H&E stain (Figure 1(b)). In the FLJ (5 mL and 10 mL) doses and silymarin treated groups, the level of vacuolization shows no differences from the CCl_4_ + H_2_O group. In the FLJ (26 mL doses) treated group, the level of liver vacuolization is higher than in the CCl_4_ + H_2_O group. In the FLJ (5 mL, 10 mL, and 26 mL doses) and silymarin treated groups, the level of liver necrosis shows no differences to the CCl_4_ + H_2_O group.

**Figure 2:**
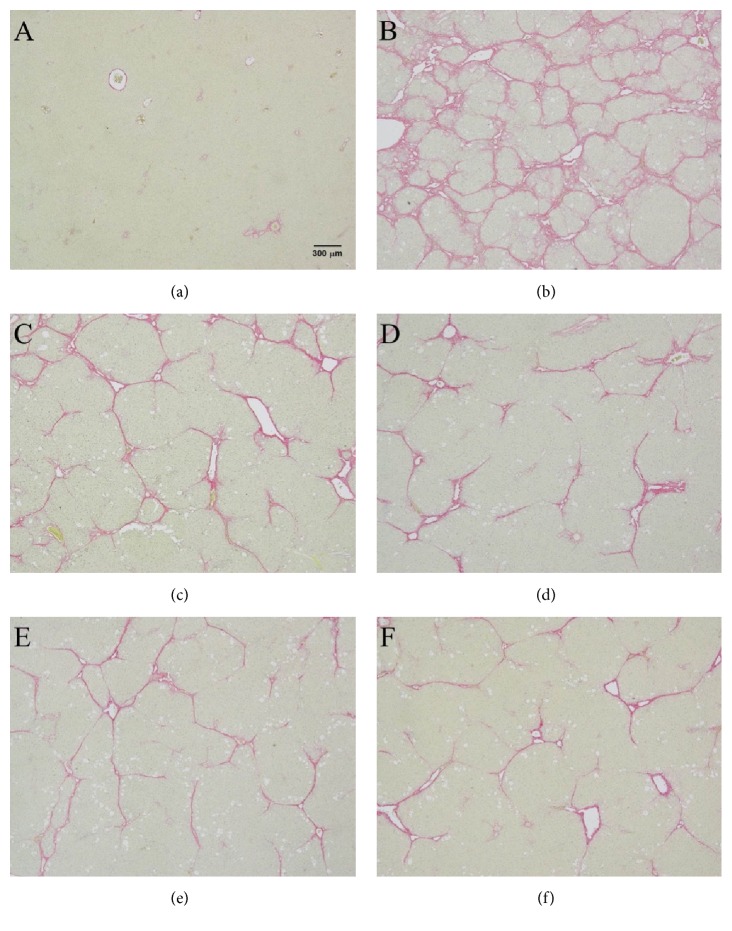
Treatments with FLJ improved the histology of CCl_4_ treated rat liver. Sirius Red staining of liver sections from (a) normal control and (b) the group that received CCl_4_ + H_2_O. Note that nodular formation and complete septa interconnecting with each other are observed; (c) CCl_4_ + FLJ 5 mL/kg group; (d) CCl_4_ + FLJ 10 mL/kg; (e) CCl_4_ + FLJ 26 mL/kg; (f) CCl_4_ + silymarin 200 mg/kg.

As Table 10 and Figure 2 show, in the livers of CCl_4_ treated rats, liver fibrosis is obviously stained by Sirius Red stain (Figure 2(b)). In FLJ (5 mL, 10 mL, and 26 mL doses) and silymarin treated groups, the level of liver fibrosis is significantly lower than in the CCl_4_ + H_2_O group.

## 4. Discussion

Hepatotoxicity induced by CCl_4_ which is a potent hepatotoxic chemical that produces free radicals is the most widely used criterion for evaluating hepatoprotective activity of plant extracts [21]. In this study, no-sugar-added fermented lemon juice (FLJ) is evaluated in terms of its anti-inflammation efficacies on liver injury caused by CCl_4_, and our data indicates the positive hepatoprotective ability of FLJ and its effectiveness comparable to silymarin, the standard drug typically prescribed to treat liver disease.

When the liver is injured, the plasma ALT and AST leak, and the activities of plasma AST and ALT increase, which are the most generally used biochemical markers of hepatitis [22]. In this study, an obvious increase in the level of AST and ALT in the serum is discovered after oral administration of CCl4. However, the increased levels of these enzymes are significantly decreased by FLJ (in 5 mL dose) oral treatments, which is comparable to silymarin (200 mg/kg dose); this indicates that the fermented lemon juice prevents liver damage, which is further confirmed by the reduced amount of histopathological injury. And it may also show the beneficial advantages of the FLJ (which are comparable to silymarin at 200 mg/kg) over dried citrus lemon, which is compared to standard silymarin at 100 mg/kg only [23].

The liver synthesizes albumin, and when the chronic liver injury causes liver fibrosis, albumin decreases [24]. In this study, the data of week 8 reveals that albumin is decreased due to the CCl_4_-induced liver injured inflammation, and then the liver soluble protein is also decreased obviously. FLJ treatments increase the albumin, and FLJ (26 mL dose) and silymarin can both increase soluble protein in liver tissues. The study proves that the declining abilities of synthesizing liver proteins caused by the CCl_4_-induced injured liver can be improved by FLJ. Liver fibrosis leads to blockage of blood flow into the liver and causes portal hypertension and also impacts blood flow to the spleen and causes splenomegaly [25]. In this study, CCl_4_-induced chronic hepatic fibrosis in rats causes splenomegaly; administration of FLJ improves splenomegaly and also shows its efficacies in reducing portal hypertension.

When the liver is damaged, it can initiate regenerative abilities [26], and thus its weight increases. However, if the liver is in a serious condition, liver atrophy shows as a consequence [27]. In this study, in CCl_4_-induced rats, hepatomegaly occurs and the level of liver water increases at the end of the experiment. After the oral treatment by FLJ (10 mL and 26 mL doses) and silymarin, the increased weights of livers of rats are reduced obviously, and the contents of liver water are decreased as well. The results show that, with FLJ treatments, the liver inflammation can be eased.

Liver fibrosis is a consequence of chronic hepatitis, and it involves irregular accumulation of extracellular matrix proteins and hyperplasticity of the connective tissue [28]. The connective tissue consists of collagen mainly, and hydroxyproline is the unique composition in collagen. The total amount of collagen can be determined by detecting the content of hydroxyproline, and it is used to express the level of liver fibrosis [29]. In this study, in CCl_4_-induced chronic hepatitis, the contents of hydroxyproline increased obviously, and with the treatments of FLJ, the content of hydroxyproline reduced as silymarin in 200 mg/kg dose does. The oral administration of FLJ also decreases the level of liver fibrosis which is further proved in the pathological examination. Histological examination using Sirius Red also shows that FLJ in different doses reduces CCl_4_-induced liver fibrosis significantly as treating with silymarin drug. The CCl_4_-induced liver fibrosis which is related to free radical and lipid peroxidation matters [30]. In this study, in CCl_4_-induced chronic hepatitis, the tissues of lipid peroxidation increase apparently, and FLJ oral administration reduces the level of hepatic lipid peroxidation. Glutathione (GSH) participates in many substantial liver cellular functions, including detoxification and free radicals scavenging and modulation of cell cycle [31, 32]. In this study, the CCl_4_ diminishes the contents of glutathione, and after FLJ treatments, the contents of glutathione rise.

Biological systems will protect themselves against injury by several means which include free radical scavengers as cellular antioxidant enzymes SOD, catalase, and GSH-Px [33]. SOD plays an important role in the exclusion of ROS derived from the peroxidative process of xenobiotics in liver tissues. Catalase is a key component of the antioxidant defense system. In the study, in CCl_4_ treated livers in rats, the activities of three liver enzymes—SOD, catalase, and GSH-Px—which assist the liver against free radicals drop. Excessive production of free radicals may result in alterations in the biological activity of cellular macromolecules [34]. With the oral administration of FLJ (by giving 3 different doses) to the CCl_4_-induced rats, the activities of these 3 enzymes have no obvious changes, and the results with comparison to silymarin and other studies reveal different outcomes from hepatoprotective effects of plants such like citrus limon, fermented mung bean, apple, and* Ginkgo biloba*, citrus maxima peel, by increasing the activities of antioxidant enzymes SOD, catalase, and GSH-Px [23, 35–38]. From the biochemical profile and histology evaluation of the study, FLJ treatment shows its hepatoprotective efficacies. The study shows a probable mechanism of action of FLJ, the observed obvious antioxidant and anti-inflammation abilities, which is different from other studies' results. This can be hypothesized from the antioxidant properties possessed by these fermented substances from FLJ, and also FLJ can increase the contents of glutathione, show its abilities to prevent liver injury caused by free radical toxicity [35].

The study strongly implies the potential use of no-added-sugar fermented lemon juice in applications for liver disease or oxidative stress prevention. Further work for isolating specific substance(s) from FLJ responsible for hepatoprotective activity and also to discover the exact mechanism of action is suggested.

## 5. Conclusions

Base on the above results, we can obtain the following conclusions from this study. The no-sugar-added fermented lemon juice (FLJ) differs from traditional fermentation using lots of sugar, which prevents hidden sugar consumption while taking it as an alternative therapy for liver inflammation, and also benefits from whole fruits with discarded peel nutrients. The level of plasma ALT and AST, hepatic lipid peroxidation, splenomegaly, and liver water are reduced significantly in rats under FLJ oral treatments, and in pathological assays of liver fibrosis, it is improved as shown in the results. Reduced hepatic injury by increasing liver soluble protein and glutathione and albumin contents is observed in FLJ treated groups. This study also reveals that FLJ has similar efficacies to silymarin. The green color and the taste of sourness are both associated with treating and healing the liver based on the five-element theory in traditional Chinese medicine, and the green and sour FLJ may be addressed to the ancient theory in preventing hepatic injury accordingly.

## Figures and Tables

**Table 1:** Weight changes of rats.

	Control	CCl_4_ + H_2_O	CCl_4_ + FLJ	CCl_4_ + FLJ	CCl_4_ + FLJ	CCl_4_ + silymarin
			5 mL/kg	10 mL/kg	26 mL/kg	200 mg/kg
Week 0	240.7 ± 6.6^a^	238.4 ± 7.6^a^	237.6 ± 8.8^a^	239.4 ± 7.0^a^	237.7 ± 7.6^a^	238.1 ± 6.8^a^
Week 1	281.3 ± 16.9^b^	268.9 ± 14.1^a^	265.2 ± 11.5^a^	267.1 ± 12.6^a^	269.5 ± 10.5^a^	266.0 ± 8.3^a^
Week 2	308.7 ± 14.8^b^	293.2 ± 14.8^a^	289.0 ± 9.3^a^	291.1 ± 10.8^a^	297.6 ± 10.5^a^	290.1 ± 8.5^a^
Week 3	356.3 ± 19.5^b^	327.8 ± 16.8^a^	326.3 ± 16.6^a^	328.3 ± 15.1^a^	332.1 ± 16.3^a^	326.5 ± 10.9^a^
Week 4	384.7 ± 23.5^b^	357.2 ± 15.3^a^	353.2 ± 22.7^a^	348.3 ± 17.4^a^	346.2 ± 32.5^a^	355.1 ± 15.3^a^
Week 5	416.0 ± 28.7^b^	384.6 ± 16.7^a^	385.5 ± 25.8^a^	380.8 ± 19.7^a^	377.3 ± 23.9^a^	376.9 ± 24.1^a^
Week 6	429.8 ± 29.4^b^	388.4 ± 17.9^a^	388.6 ± 26.9^a^	386.3 ± 19.5^a^	381.2 ± 28.4^a^	380.8 ± 22.0^a^
Week 7	453.6 ± 33.0^b^	404.9 ± 21.3^a^	412.4 ± 30.8^a^	404.0 ± 17.8^a^	397.5 ± 30.0^a^	401.3 ± 29.8^a^
Week 8	464.4 ± 36.3^b^	408.6 ± 27.2^a^	420.1 ± 29.2^a^	412.6 ± 19.4^a^	408.3 ± 33.6^a^	408.9 ± 28.4^a^

All values are means ± SD (10–12). a and b represent the results; *P* value < 0.05 is considered statistically significant.

**Table 2:** Effect of FLJ on the plasma AST, ALT, and albumin in CCl_4_-induced rats.

Remedies		Control	CCl_4_ + H_2_O	CCl_4_ + FLJ	CCl_4_ + FLJ	CCl_4_ + FLJ	CCl_4_ + silymarin
		-	-	5 mL/kg	10 mL/kg	26 mL/kg	200 mg/kg
Week 1	AST (U/L)	72.7 ± 6.2^a^	329.0 ± 98.6^c^	184.2 ± 71.5^b^	160.5 ± 41.0^b^	319.1 ± 82.1^c^	224.3 ± 100.6^b^
	ALT (U/L)	44.4 ± 4.1^a^	163.8 ± 59.2^c^	93.6 ± 13.4^b^	83.8 ± 24.9^b^	158.3 ± 46.2^c^	111.6 ± 67.2^b^

Week 3	AST (U/L)	62.6 ± 7.6^a^	725.9 ± 319.5^c^	467.7 ± 338.2^b^	516.5 ± 151.8^b^	483.4 ± 106.3^b^	451.3 ± 194.9^b^
	ALT (U/L)	37.6 ± 2.5^a^	398.3 ± 194.1^c^	242.8 ± 162.1^b^	288.7 ± 105.8^bc^	279.3 ± 117.1^bc^	258.4 ± 134.6^b^

Week 6	AST (U/L)	68.7 ± 9.1^a^	2195.5 ± 1348.6^c^	1270.5 ± 442.1^b^	897.3 ± 306.8^b^	1307.5 ± 555.9^b^	1328.5 ± 738.8^b^
	ALT (U/L)	44.2 ± 4.0^a^	1793.3 ± 878.6^c^	1055.7 ± 285.5^b^	795.6 ± 274.9^b^	1063.0 ± 449.5^b^	1030.0 ± 648.6^b^

Week 8	AST (U/L)	75.6 ± 9.1^a^	3363.0 ± 986.2^c^	1745.5 ± 832.1^b^	1754.0 ± 891.2^b^	1764.0 ± 1189.6^b^	1606.9 ± 838.1^b^
	ALT (U/L)	43.7 ± 10.2^a^	2960.8 ± 1014.4^c^	1943.6 ± 613.4^b^	1742.5 ± 776.3^b^	1492.5 ± 621.8^b^	1487.3 ± 667.2^b^
	Albumin (g/dL)	3.64 ± 0.21^c^	2.96 ± 0.38^a^	3.29 ± 0.18^b^	3.29 ± 0.24^b^	3.52 ± 0.33^bc^	3.26 ± 0.49^b^

All values are means ± SD (10–12). a, b, and c represent the results; *P* value < 0.05 is considered statistically significant.

**Table 3:** Effects of liver weights of CCl_4_-induced injured liver in rats.

Remedies	Doses (mg/kg)	Liver (g)	Liver (%)	Liver water (%)
Control	-	11.6 ± 1.1^a^	2.5 ± 0.2^a^	69.6 ± 1.2^a^
CCl_4_ + H_2_O	-	18.2 ± 1.7^d^	4.5 ± 0.5^c^	73.2 ± 1.0^c^
CCl_4_ + FLJ	5	17.7 ± 2.5^cd^	4.2 ± 0.7^bc^	71.8 ± 1.3^b^
	10	15.7 ± 1.3^b^	3.8 ± 0.3^b^	71.7 ± 1.4^b^
	26	16.4 ± 2.2^bc^	4.1 ± 0.7^bc^	71.6 ± 1.7^b^
CCl_4_ + silymarin	200 mg/kg	15.3 ± 2.0^b^	3.8 ± 0.6^b^	72.2 ± 2.0^bc^

All values are means ± SD (10–12). a, b, c, and d represent the results; *P* value < 0.05 is considered statistically significant.

**Table 4:** Effects of spleen weights of CCl_4_-induced injured liver in rats.

Remedies	Doses (mL/kg)	Spleen (g)	Spleen (%)
Control	-	0.89 ± 0.08^a^	0.19 ± 0.01^a^
CCl_4_ + H_2_O	-	1.67 ± 0.25^d^	0.41 ± 0.07^c^
CCl_4_ + FLJ	5	1.27 ± 0.18^c^	0.30 ± 0.04^b^
	10	1.17 ± 0.24^bc^	0.28 ± 0.05^b^
	26	1.13 ± 0.24^bc^	0.28 ± 0.07^b^
CCl_4_ + silymarin	200 mg/kg	1.06 ± 0.25^ab^	0.26 ± 0.07^b^

All values are means ± SD (10–12). a, b, c, and d represent the results; *P* value < 0.05 is considered statistically significant.

**Table 5:** The effects of glutathione and malondialdehyde.

Remedies	Doses (mL/kg)	Glutathione (*μ*mol/g tissue)	Malondialdehyde (nmol/mg protein)
Control	-	2.9 ± 0.4^b^	4.3 ± 1.3^a^
CCl_4_ + H_2_O	-	2.4 ± 0.3^a^	5.9 ± 1.2^c^
CCl_4_ + FLJ	5	2.8 ± 0.5^b^	4.9 ± 0.9^a^
	10	2.8 ± 0.3^b^	4.6 ± 0.7^a^
	26	2.8 ± 0.4^b^	4.4 ± 1.0^a^
CCl_4_ + silymarin	200 mg/kg	2.8 ± 0.6^b^	4.7 ± 1.2^a^

All values are means ± SD (10–12). a, b, and c represent the results; *P* value < 0.05 is considered statistically significant.

**Table 6:** The effect of protein and hydroxyproline.

Remedies	Doses (mL/kg)	Protein (mg/g tissue)	Hydroxyproline (*μ*g/g tissue)
Control	-	159.9 ± 32.1^b^	175.6 ± 43.9^a^
CCl_4_ + H_2_O	-	121.5 ± 22.2^a^	352.3 ± 30.1^c^
CCl_4_ + FLJ	5	140.3 ± 31.5^ab^	300.8 ± 47.4^b^
	10	148.8 ± 32.9^ab^	286.8 ± 38.6^b^
	26	153.2 ± 35.5^b^	281.5 ± 61.9^b^
CCl_4_ + silymarin	200 mg/kg	150.2 ± 38.5^ab^	309.8 ± 41.8^b^

All values are means ± SD (10–12). a, b, and c represent the results; *P* value < 0.05 is considered statistically significant.

**Table 7:** The effects of SOD, catalase, and GSH-Px activities.

Remedies	Doses (mL/kg)	SOD (U/mg protein)	Catalase (U/mg protein)	GSH-Px (mU/mg protein)
Control	-	36.6 ± 4.7^b^	11.4 ± 2.0^b^	1942.1 ± 464.6^c^
CCl_4_ + H_2_O	-	24.2 ± 4.7^a^	8.6 ± 2.7^a^	598.9 ± 118.5^a^
CCl_4_ + FLJ	5	26.5 ± 6.2^a^	10.5 ± 2.5^ab^	764.7 ± 125.96^a^
	10	26.1 ± 5.6^a^	9.2 ± 2.6^ab^	782.8 ± 198.8^a^
	26	25.5 ± 7.1^a^	10.1 ± 2.1^ab^	777.1 ± 175.6^a^
CCl_4_ + silymarin	200 mg/kg	28.9 ± 6.3^a^	9.3 ± 2.46^ab^	1085.4 ± 405.6^b^

All values are means ± SD (10–12). a, b, and c represent the results; *P* value < 0.05 is considered statistically significant.

**Table 8:** Effects of liver vacuolization.

Remedies	Doses (mL/kg)	Vacuolization					
		0	1	2	3	4	Average
Control	-	10	0	0	0	0	0 ± 0^a^
CCl_4_ + H_2_O	-	0	3	4	4	1	2.3 ± 1.0^b^
CCl_4_ + FLJ	5	0	2	6	4	0	2.2 ± 0.7^b^
	10	0	1	7	3	1	2.3 ± 0.8^bc^
	26	0	0	3	7	2	2.9 ± 0.7^c^
CCl_4_ + silymarin	200 mg/kg	0	1	7	4	0	2.3 ± 0.6^b^

Grade designations: (0) normal; (1) very slight; (2) slight; (3) moderate; (4) severe. Each value is the number of rats with grading changes. (*P* > 0.05). All values are means ± SD (10–12). a, b, and c represent the results; *P* value < 0.05 is considered statistically significant.

**Table 9:** Effects of liver necrosis.

Remedies	Doses (mL/kg)	Necrosis					
		0	1	2	3	4	Average
Control	-	10	0	0	0	0	0 ± 0^a^
CCl_4_ + H_2_O	-	0	6	3	3	0	1.8 ± 0.9^bc^
CCl_4_ + FLJ	5	0	7	4	1	0	1.5 ± 0.7^b^
	10	0	6	5	1	0	1.6 ± 0.7^b^
	26	0	1	8	3	0	2.2 ± 0.6^c^
CCl_4_ + silymarin	200 mg/kg	0	6	6	0	0	1.5 ± 0.5^b^

Grade designations: (0) normal; (1) very slight; (2) slight; (3) moderate; (4) severe. Each value is the number of rats with grading changes. (*P* > 0.05). All values are means ± SD (10–12). a, b, and c represent the results; *P* value < 0.05 is considered statistically significant.

**Table 10:** Effects of liver fibrosis.

Remedies	Doses (mL/kg)	Fibrosis					
		0	1	2	3	4	Average
Control	-	10	0	0	0	0	0 ± 0^a^
CCl_4_ + H_2_O	-	0	0	2	3	7	3.4 ± 0.8^c^
CCl_4_ + FLJ	5	0	0	7	3	2	2.6 ± 0.8^b^
	10	0	4	3	3	2	2.3 ± 1.1^b^
	26	0	2	5	5	0	2.3 ± 0.8^b^
CCl_4_ + silymarin	200 mg/kg	0	1	8	2	1	2.3 ± 0.8^b^

Grade designations: (0) normal; (1) very slight; (2) slight; (3) moderate; (4) severe. Each value is the number of rats with grading changes. (*P* > 0.05). All values are means ± SD (10–12). a, b, and c represent the results; *P* value < 0.05 is considered statistically significant.
